# Establishment of reference CD4+ T cell values for adult Indian population

**DOI:** 10.1186/1742-6405-8-35

**Published:** 2011-10-03

**Authors:** Madhuri R Thakar, Philip R Abraham, Sunil Arora, Pachamuthu Balakrishnan, Bhaswati Bandyopadhyay, Ameeta A Joshi, K Rekha Devi, Ravi Vasanthapuram, Madhu Vajpayee, Anita Desai, Janardhanan Mohanakrishnan, Kanwar Narain, Krishnangshu Ray, Shilpa S Patil, Ravinder Singh, Anuj Singla, Ramesh S Paranjape

**Affiliations:** 1 Department of Immunology, National AIDS Research Institute, G-73, MIDC, Bhosari, Pune, 411026, India; 2 Department of Immunopathology, Postgraduate Institute of Medical education & Research, Sector:12, Chandigarh-160012, India; 3 Infectious Diseases Laboratory, YRG Centre for AIDS Research and Education, Rajiv Gandhi Salai, Taramani, Chennai-600113, India; 4 Department of Virology, School of Tropical Medicine, C.R.Avenue, Kolkata-700073, West Bengal, India; 5 Department of Microbiology, Grant Medical College & Sir J J Hospital, Byculla, Mumbai-400008, India; 6 Division of Enteric Diseases, Regional Medical Research Centre, N.E.Region, Indian Council of Medical Research, Dibrugarh-786001, Assam, India; 7 Department of Neurovirology, National Institute of Mental Health and Neuro Sciences Hosur Road, Bangalore-560029, India; 8 Laboratory Head, HIV & Immunology division, Department of Microbiology,All India Institute of Medical Sciences, Ansari Nagar, New Delhi-110029 India; 9 Department of Clinical and Experimental Pharmacology, School of Tropical Medicine, C.R.Avenue, Kolkata-700073, West Bengal, India

## Abstract

**Background:**

CD4+ T lymphocyte counts are the most important indicator of disease progression and success of antiretroviral treatment in HIV infection in resource limited settings. The nationwide reference range of CD4+ T lymphocytes was not available in India. This study was conducted to determine reference values of absolute CD4+ T cell counts and percentages for adult Indian population.

**Methods:**

A multicentric study was conducted involving eight sites across the country. A total of 1206 (approximately 150 per/centre) healthy participants were enrolled in the study. The ratio of male (N = 645) to female (N = 561) of 1.14:1. The healthy status of the participants was assessed by a pre-decided questionnaire. At all centers the CD4+ T cell count, percentages and absolute CD3+ T cell count and percentages were estimated using a single platform strategy and lyse no wash technique. The data was analyzed using the Statistical Package for the Social Scientist (SPSS), version 15) and Prism software version 5.

**Results:**

The absolute CD4+ T cell counts and percentages in female participants were significantly higher than the values obtained in male participants indicating the true difference in the CD4+ T cell subsets. The reference range for absolute CD4 count for Indian male population was 381-1565 cells/μL and for female population was 447-1846 cells/μL. The reference range for CD4% was 25-49% for male and 27-54% for female population. The reference values for CD3 counts were 776-2785 cells/μL for Indian male population and 826-2997 cells/μL for female population.

**Conclusion:**

The study used stringent procedures for controlling the technical variation in the CD4 counts across the sites and thus could establish the robust national reference ranges for CD4 counts and percentages. These ranges will be helpful in staging the disease progression and monitoring antiretroviral therapy in HIV infection in India.

## Introduction

CD4 + T lymphocytes play a central regulatory role in the immune response. The decrease in CD4+ T cell numbers can compromise the normal immune functions of the body. The number of CD4+ T cells in circulation provides important information about the immune competence of an individual. Hence estimation of CD4+ T cells is an important parameter in immune deficiency disorders. The clinical applications of immunophenotyping of CD4+ T cells include the diagnosis of immunodeficiency disorders, the evaluation of immune-mediated diseases, the assessment of immune reconstitution following stem cell transplantation and the monitoring of disease progression in Human Immunodeficiency Virus (HIV) infection.

HIV infects CD4+ T lymphocytes selectively and causes the destruction of CD4+ T cells directly as well as indirectly leading to gradual loss of the CD4+ T cell numbers in peripheral circulation. Hence, the CD4+ T cell counts are being used to monitor the disease progression in HIV infection, to decide the threshold for initiation of anti-retro viral therapy, to monitor the efficacy of ART and to initiate prophylactic treatment for opportunistic infections (OIs) [1,2].

The CD4+ T cell counts are known to be influenced by race and environmental factors [3-9]. Hence, it is important to establish the reference ranges for the CD4+ T cell counts in the target population to understand the extent of immune dysfunction. The information on the lower limits of the CD4+ T cell count in normal healthy population is necessary to decide the threshold for initiation and monitoring of ART. India is one of the countries affected significantly by HIV and has approximately 2.4 million people living with HIV/AIDS [10]. Some of the recent studies have shown that the treatment at higher CD4+ T cell numbers may be beneficial in reducing the clinical events and death. WHO has already recommended increase of threshold for initiating ART to 350 cells/μL. It is important to determine the reference CD4+ T cell values to ensure that ART is not initiated when CD4+ T cell counts are in normal range, in case the threshold is increased further. No such ranges are available for Indian population based on a multi-centric well designed study. Many of the published Indian studies had inadequate sample size and hence, the results from these studies cannot be generalized. Only one study presented data with adequate sample size that included populations from different parts of the country, however this study established only the reference range for CD4+ T cell percentages and not for absolute counts [11]. The comparison of the published data on the CD4+ T cells count in healthy Indian adult population and other parts of the world showed that the CD4+ T cell counts differ with the population, suggesting that each population should have its own reference range for lymphocyte subsets [12].

This report presents data from a multicentric study carried out to define the reference ranges of the absolute CD4 + T cell counts and CD4+ T cell percentages from healthy Indian adults using a standard protocol to reduce the variability in both sample preparation and flow cytometer operation. The influence of sex, age, economic status, marital status, smoking, alcohol consumption on CD4+ T cell count was also evaluated.

## Methods

### Study participants

The study was conducted at eight centers across four geographical zones of the country; Post Graduate Institute of Medical Education & Research, Chandigarh and All India Institute of Medical Sciences, New Delhi from the north zone; National AIDS Research Institute, Pune and JJ Hospitals and Grant Medical College, Mumbai from the west zone; Y.R. G Centre for AIDS Research and Education, Chennai and National Institute of Mental Health and Neuro Sciences, Bengaluru from the south zone and School of Tropical Medicine, Kolkata and Regional Medical Research Centre, Dibrugarh from the east zone. A total of 1206 healthy individuals (approximately 150/center and 300/region) were enrolled. The institutional Ethics Committees of all the centers approved the study. The required information and blood samples were collected after obtaining written informed consent.

The participants 18 years of age or older were enrolled in the study. The healthy status of the participants was assessed by a pre-decided questionnaire which included information on history of recent (within the last 3 months) episode of fever, rigors, sweats, fatigue, weight Loss, diarrhoea, the information on the history of tuberculosis and asthma. Additionally, the history of vaccination, blood transfusion, allergy, and major surgery was obtained to assess their healthy status. The participants giving history of the recent enteric, respiratory infections and malaria were excluded. Participants with symptoms, investigations and treatment suggestive of tuberculosis, with history of vaccination or blood transfusion within last one year, autoimmune diseases were excluded. The female participants with pregnancy were also excluded. The data was collected on age, sex, marital status, occupation, economic status (as self graded family income), place of origin and habits like smoking and alcohol consumption using the modified scale [13,14].

### Sample collection and processing

Five mL of Whole blood sample was collected in K3EDTA evacuated tubes between 9 AM to 12 noon from all participants at all sites. The samples were processed for CD4+ T cell count estimation on the same day. The results were entered in a structured database.

### CD4 count estimation

The CD4+ T cell count estimation was carried out using the standard flow cytometers adapted to single platform technology. The FACSCalibur (Becton Dickinson, USA) was used by seven participating centers except once from South India. This center used EPIX XL-MCL (Beckman Coulter Inc, CA, USA).

For the FACSCalibur, 20 μL of liquid antibody reagent (MultiTEST CD3 FITC, CD4 PE, CD45 PerCP, Cat no.340402, Becton Dickinson, USA) and 50 μL of whole blood was added to the TruCOUNT tube (Cat No: 340334, Becton Dickinson) containing the reference beads. The tube was vortexed and incubated at ambient temperature in dark for 15 minutes. The RBCs were lysed using 450 μl of 1:10 diluted lysing solution (FACSlysing solution, Cat No: 349202, Becton Dickinson, USA) for 15 minutes in the dark at ambient temperature. The stained sample was acquired on flowcytometer (FACSCalibur, Becton Dickinson, USA) and analyzed using the MultiSET software (BD Biosciences). In brief, the CD3+, CD4+ T cell populations were identified using the logarithmic amplification of fluorescence parameters (FL1, FL2, FL3 i.e. FITC, PE and PerCP fluorescent dye conjugated with monoclonal antibodies against CD3, CD4 and CD45 respectively). Forward scatter and side scatter (SSC-H) signals were measured using a linear scale. After acquiring data up to 100, 000 events per sample, a gate (R1) was automatically set on the lymphocyte cluster (SSC-H low/CD45 PerCP high+ cells). The second two-parameter dot plot (FL1 vs FL2) was automatically generated showing double positive (CD3+/CD4+) T cells in upper right region. Percent and absolute counts of CD4+, CD3+ T lymphocytes were then generated by the MultiSET software.

For the EPICS XL-MCL, 10 μL of liquid antibody reagent (CYTO-STAT^® ^triCHROME™ CD8-FITC/CD4-RD1/CD3-PC5, Beckman Coulter Inc.) and 100 μL of whole blood was added to the Coulter tube and incubated in the TQ-Prep (Beckman Coulter Inc., CA, USA) for 10 minutes. The TQ-Prep automatically adds the lysis, stabilizer and fixative to the tubes, to which 100 μL of Flowcount (Beckman Coulter Inc.) is added manually. The tube was vortexed and analyzed with EPICS XL-MCL. Forward scatter and side scatter (SS) signals were measured using a linear scale to gate the lymphocytes. From the gated lymphocyte population, the CD4+ T-cell populations were identified using the logarithmic amplification of fluorescence parameters (FL2 and FL4, i.e. PE and PC5 fluorescent dyes conjugated with monoclonal antibodies against CD4 and CD3, respectively). After the acquisition of events, a gate was automatically set on the lymphocyte cluster (SS/FS). The two-parameter dot plot (FL4 vs FL2) was automatically generated showing double positive (CD3+/CD4+) T-cells in upper right region. Percent and absolute counts of CD4+ T lymphocytes were then generated by the System II software.

### Quality control

To ensure the optical alignment of the equipment and fluorescence compensation settings, the calibration beads (CaliBRITE™3) were run every day and the compensation was assessed using the FACSComp software for FACSCalibur. While for EPICS XL-MCL, the alignment and compensation was ensured using the Flow-Check fluorospheres and the data was analyzed using Flow-Set beads each day before running of samples. As an internal quality control, all the centers ran the commercially available stabilized blood samples before the samples and the samples were run only when the controls showed less than 10% variation as compared to the values obtained in the previous run. All the centers successfully participated (3 times per year) in external proficiency testing programme run either by international program for Quality Assessment and Standardization for Immunological Measures(QASI) from Canada or the United Kingdom National External Quality Assurance Service (UKNEQAS) from United Kingdom for lymphocyte subset estimation.

The variation between the CD4+ T cell count and percentages obtained using the two methodologies (FACSCalibur and EPICS XL-MCL) was assessed using the same stabilized blood samples tested by both the machines at two different centers. These samples included 30 replicates of Immuno Trol (Lot# 7587024, Beckman Coulter, USA) and samples received under the proficiency programme from UKNEQAS (N = 22). The%CV and correlation coefficient(r) was calculated for both the categories of the samples. The results are given in the table 1. The variation (%CV) between the values obtained for absolute CD4 counts was 5.17% and 7.19% for the Immuno Trol and proficiency samples respectively whereas for CD4% the %CV was 7.04% and 4.6% for the Immuno Trol and proficiency samples respectively. The correlation coefficient (r) ranged between 0.891 and 0.993 for all the values.

**Table 1:** Inter equipment comparison between FACSCalibur and EPICS XL-MCL using the stabilized blood samples

	Immuno Trol Lot# 7587024 (N = 30)		UKNEQAS proficiency samples(N = 22)	
	r value	%CV	r value	%CV

Absolute CD4 count(cells/μL)	0.917	5.17	0.975	7.19

CD4 percentage	0.891	7.04	0.993	4.6

The inter equipment comparison between FACSCalibur and EPICS XL-MCL was conducted using the stabilized blood samples. The r values (range: 0.89 to 0.99) were acceptable. The % CV (range: 4.6 to 7.1) were > 10% and hence acceptable.

### Data analysis

The data was analyzed using the Statistical Package for the Social Scientist (SPSS, version 15) and Prism software version 5. Patients were stratified into the respective geographical zones on the basis of their place of origin before the analysis. The mean, median and standard deviation values were calculated for absolute CD4 + T cell count and percentages. The frequency of distribution of absolute CD4+ T cell count and percentages was analyzed for each geographical region. The result of the Wilks-Shapiro test for normality was significant, the data were considered to have a non-Gaussian distribution, and the reference range was defined as the central 95% of the area under the distribution curve of values (i.e. from 2.5 to 97.5%) Differences between genders were estimated using the Mann-Whitney U test was used to estimate the differences between the CD4 counts and percentages among the individuals with different demographics such as gender, age, nutrition, marital status, economic status and habits such as alcohol use, and smoking. P values less than 0.05 were considered to be significant for all analyses.

## Results

### Demographic characteristics

A total of 1206 healthy participants were enrolled in the study with a ratio of male (N = 645) to female (N = 561) of 1.14:1. Of 1206, 305 subjects belonged to the eastern region, 302 to western, 299 to northern and 300 to Southern region of India. The ratio of male to female ranged in each region from 1:1 to 1.5: 1. Ninety nine percent participants were from age group of 18-50 years. The mean age of male (31 years, range: 17-72) and female (31 years, range: 18-59) participants were similar. Of these 1206 subjects, 723 were married and 428 were unmarried or single. For the remaining participants, the status was not known. Of the 1206 participants, 75% reported medium level income (Rs. 15000-25000/month), 17% low level income(less than Rs. 15000/month) and 8% reported high level income (more than Rs. 25000/month). Only 5 subjects were habitual alcohol consumers (more than 21 measures/week), 108 were occasional (1-21 measures/week) where as 1091 reported no alcohol consumption. Similarly, 29 were habitual smokers (more than 5/day), 60 were occasional (1-5/day) and 1115 did not smoke at all.

### Absolute CD4+ T cell counts

**Figure 1:**
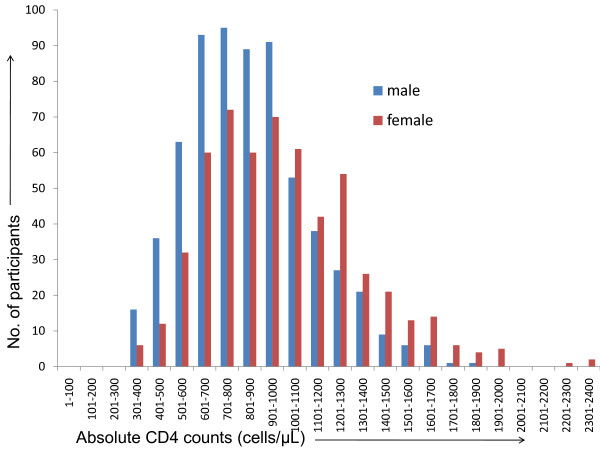
**The figure showed the frequency distribution of absolute CD4 counts in healthy Indian adults**. The × axis shows the absolute CD4 counts and the Y axis shows the number of participants. The blue and red bar represents the number of male and female participants respectively.

The overall mean absolute CD4+ T cell count was 919 ± 312 cells/μL (median 877, range: 302-2371 cells/μL). Ninety eight percent of the study population showed absolute CD4 counts between 400-2000 cells/μL (Figure 1). The 17 male and 6 female participants showing CD4 counts less than 400 cells/μL are distributed throughout the four geographical regions and not confounded to any particular region. The absolute CD4 + T cell counts in female (mean: 995 ± 335, median: 953 cells/μL) were significantly higher than CD4+ T cell counts in male (mean: 852 ± 273, median: 822 cells/μL) participants. The region wise absolute CD4+ T cell counts from male and female study participants are given in table 2. The values were significantly higher in female than male participants in all the regions. Also there was significant difference amongst the mean CD4+ T cell counts of the female participants from all four geographical regions (p < 0.05). Whereas the CD4 counts of male participants from North and East regions and from South and West regions did not differ significantly. The absolute CD4 + T cell counts of the participants from Southern region were significantly higher than the CD4 counts of the participants from the other regions with the lowest CD4+ T cell counts in the participants from the Eastern region (Table 2). Since the normal distribution could not be achieved for absolute CD4 counts (both region and gender wise), the 2.5% and 97.5% percentile covering 95% of the population was considered to be the reference range for absolute CD4+ T cell counts. The reference range for absolute CD4+ T cell count for Indian male population was 381-1565 cells/μL and for female population was 447-1846 cells/μL.

**Table 2:** The absolute CD4 count and percentages in the adult Indian population

	Male				Female					Total			
**Region**	**N**	**Mean ± SD**	**Median**	**2.5^th ^-97.5^th ^percentile**	**N**	**Mean ± SD**	**Median**	**2.5^th ^-97.5^th ^percentile**	**P value**	**N**	**Mean ± SD**	**Median**	**2.5^th ^-97.5^th ^percentile**
**Absolute CD4 count (cells/μL)**													
**East**	**187**	782 ± 249	756	352-1387	**118**	827 ± 267	759	418-1587	0.02	**305**	808 ± 255	773	426-1367
**West**	**160**	890 ± 280	853	452-1602	**142**	1020 ± 323	964	499-1916	0.002	**302**	952 ± 307	918	484-1646
**North**	**156**	825 ± 271	778	354-1551	**143**	951 ± 343	907	368-1895	0.0009	**299**	885 ± 316	831	381-1590
**South**	**142**	931 ± 271	931	389-1649	**158**	1139 ± 322	1116	586-1977	< 0.0001	**300**	1039 ± 315	1017	538-1701
**Total**	**645**	852 ± 273	822	381-1565*	**561**	995 ± 335	953	447-1846*	< 0.0001	**1206**	919 ± 311	877	**448-1611**
**CD4 Percentage**													
**East**	**187**	35 ± 6	35	24-48	**118**	38 ± 6	37	27-50	0.001	**305**	36 ± 6	36	25-48
**West**	**160**	37 ± 6	37	25-48	**142**	40 ± 7	40	28-54	< 0.0001	**302**	38 ± 7	38	27-50
**North**	**156**	37 ± 6	36	27-51	**143**	39 ± 6	39	28-54	0.0011	**299**	38 ± 6	38	27-52
**South**	**142**	36 ± 6	36	25-48	**158**	40 ± 6	40	28-54	< 0.0001	**300**	38 ± 7	38	27-52
**Total**	**645**	36 ± 6	36	25-49*	**561**	39 ± 6	39	27-54*	< 0.0001	**1206**	38 ± 7	37	**27-51**

The table showed the mean, median and 2.5^th ^-97.5^th ^percentile values of the absolute CD4 counts and percentages form the adult Indian population. The region wise and gender wise values of CD4 counts and percentages have been given. The absolute CD4 counts and percentages form female and male participants were compared using the Mann Whitney's non parametric t test and found to be significantly higher in female participants.

*: The reference ranges as shown by 2.5^th ^-97.5^th ^percentile have been mentioned in bold.

### CD4 + T cell percentage

The overall mean CD4 percentage was 38% (median 37%, range: 14-60%). The CD4 percentages were significantly higher in female participants (mean ± SD: 39 ± 6%, median 39%) than male participants (mean ± SD: 36 ± 6%, median 36%). The CD4 percentages were significantly higher in female in all the regions (Table 2). When the region wise data on CD4% was analysed, the CD4% from the eastern region (mean CD4%: 35 ± 6, median: 35%) was significantly lower than the CD4% from other parts of the country (mean CD4%: 36 ± 6 and 37 ± 6, median: 36% and 37%) The 2.5% and 97.5% covering 95% of the population was considered to be the reference range for CD4 percentages. The reference range for CD4% for Indian male population was 25-49% and for female population was 27-54%.

### Absolute CD3 count and percentages

The overall mean absolute CD3+ T cell count was 1692 ± 548 cells/μL (median 1628, range: 457-3926 cells/μL) and the mean CD3 percentage was 69 ± 6.9% (median 69%, range: 55-81%). The absolute CD3 + T cell counts and percentages in female participants were significantly higher than CD3+ T cell counts in male (mean absolute CD3 counts: 1769 ± 571 in female and 1625 ± 516 in male, p < 0.0001 and mean CD3%: 70 ± 7 in female and 67 ± 7.2% in male participants, p < 0.001). The CD3 counts of the participants from Southern region (mean: 1899+508, median: 1860 cells/μL) were significantly higher than the CD3 counts of the participants from the other regions with the lowest CD3+ T cell counts in the participants from the Eastern region(mean: 1504 ± 500, median: 1418 cells/μL). Whereas the CD3% from the eastern region (mean: 67+7.2, median: 68%) was significantly lower than the CD3% from other parts of the country (mean: 70 ± 6.5, 70 ± 6.6 and 70 ± 6.9, median: 70%). The reference range (2.5% and 97.5% percentile covering 95% of the population) for absolute CD3+ T cell count for Indian male population was 776-2785 cells/μL and for female population was 826-2997 cells/μL. The reference range for CD3% for Indian male population was 54-81% and for female population was 56-81%.

### Influence of the social, demographic and other factors on CD4 count and percentages

The absolute CD4+ T cell counts and percentages did not differ significantly in the individuals with habitual, occasional or no alcohol consumption (p > 0.05 within all groups). The smoking habits of the enrolled participants did not seem to influence the absolute CD4+ T cell counts and percentages (p > 0.05, for all groups) (Table 3). The diet habits (vegetarian or non-vegetarian) and marital status did not show any influence, however the income had shown to influence the absolute CD4 + T cell counts and percentages. The CD4 + T cell counts and percentages were significantly higher in individuals with high income (mean and median absolute CD4 count: 1168 and 1167 cells/μL respectively, mean and median CD4%: 40%) as compared to the participants reported to have medium level income (mean absolute CD4 count: 931 and median: 858 cells/μL, mean and median CD4%: 38%) and low level income (mean absolute CD4 count: 734 and median: 781 cells/μL, mean and median CD4%:36%) (Table 3). The absolute CD3 counts and percentages were not influenced by the alcohol consumption, smoking, diet habits and income (P > 0.05 in all cases)

**Table 3:** Influence of Age, Income status, smoking and alcohol consumption on absolute CD4 count and percentages

	N	CD4 count(Cells/μL)			CD4 percentage		
		Mean ± SD	Median	P value	Mean ± SD	Median	P value
**Age**							

< 18	1	1728	1728	----	52	52	---
18-24	270	911 ± 306	873		38 ± 7	38	
25-40	766	895 ± 295	842	< 0.001	37 ± 6	37	0.01
41-60	168	1034 ± 357	994		39 ± 7	38	
≥ 61	1	1201	1201	---	34	34	----

**Marital status**							

Married	723	924 ± 326	876	0.706	38 ± 6	37	0.828
Unmarried/Single	428	916 ± 301	878		38 ± 6	37	

**Income status**							

High	102	1168 ± 368	1167	< 0.001	40 ± 7	40	< 0.001
Medium	900	931 ± 285	858		38 ± 6	38	
Low	204	734 ± 289	781		36 ± 7	36	

**Alcohol Consumption**							

Habitual	5	770 ± 232	806	> 0.05	37 ± 10	37	> 0.05
Occasional	108	924 ± 278	900		38 ± 7	37	
No	1091	919 ± 315	873		38 ± 6	37	

**Smoking**							

Habitual	29	958 ± 274	924	> 0.05	37 ± 7	38	> 0.05
Occasional	60	908 ± 316	861		38 ± 7	37	
No	1115	919 ± 312	877		38 ± 6	37	

The influence of age, marital and income status and habits like smoking and alcohol consumption on CD4 counts was assessed using the Mann Whitney's U test. The p values less than 0.05 considered to be significant.

## Discussion

We have established the national reference range for CD4+ T cell subset, both for CD4 percentages and absolute CD4+ T cell counts, using the uniform protocol at eight centres across the country and enforcing strict quality control for a single-platform method. We have used the lyse-no wash procedure for CD4+ T cell count estimation which has been reported to control the inter laboratory variation [15]. This has reduced the chances of the variability. All the seven sites used FACSCalibur for absolute CD4 count enumeration while the EPIC XL flow cytometer from Coulter was used at one centre from South India. Before initiation, the inter equipment variability between these two equipment was determined using the stabilized blood samples. The percent CV was found to be very good, below 10%. The variability was also determined during the study using the samples received from UKNEQAS for proficiency and the %CV was found to be less than 10% making the results from both the equipments comparable. The blood sample was uniformly collected in the morning to reduce the diurnal variation as the diurnal variation is reported to add as high as 20% variation in the CD4+ T cell counts [16].

Since India is a heterogeneous country, similar number of participants from all four geographical areas was ensured. The representation from all adult age groups was also ensured. Hence, this multicentric study covering the entire country gave the opportunity to assess the differences in CD4+ T cell counts in different populations in India. The sample size of the study was in accordance with the rigorous CLSI guidelines for determining laboratory reference ranges, which recommends a minimum of 153 subjects for a 95^th ^percentile clinical reference range determination with 95% confidence [17].

The values of CD4 counts obtained in the present study were compared with the values reported in from other parts of the world (Table 4). The overall mean absolute CD4+ T cell counts and percentages obtained in the study were comparable to the values reported from Caucasian populations [18,7], British [19] and Dutch populations [6], Thai population (910 ± 310 cells/μL) [20] and population from Nigeria (861 cells/μL) [21].

**Table 4:** CD4 counts in normal healthy individuals from different countries/populations

Country/study population	Mean Absolute CD4 count (cells/mm^3^)	Reference
Caucasian	1036	18

Caucasian	868	7

United kingdom	830	19

Dutch	993	6

Thais	910	20

Nigeria	861	21

Chinese population	727	22

Tanzania	746, median 723	23

Botswana	759, median 726	24

Uganda	1256	25

Turkey	1095, median: 1055	26

India	Male: mean: 852 ± 273, median: 822 Female: mean: 995 ± 335, median: 953	Present study

The mean absolute CD4 counts obtained in different populations or countries have been summarized in the table. The mean and median CD4 counts if available have been given in the table and the last column gives the reference in which the data has been published.

The values obtained in present study were higher than the absolute CD4+ T cell counts of Chinese population (median 730 cells/μL) [22] and the values reported from a few African countries such as Tanzania (median 723 cells/μL) [23], and Botswana (median 726 cells/μL) [24]. Whereas higher CD4 counts have been reported in adult healthy populations from Uganda [25] and Turkey [26] as compared to the CD4 counts reported in the present study. These differences might be due to the racial and ethnic variations within the populations and also due to the differences in the methodologies where the variations were not controlled. The use of standardized procedures, calibrated controls and successful participation of all participating centres in external quality assurance programmes in the present study has made it possible to control the variations and generate robust reference values.

Both the absolute CD4+ T cell counts and percentages were significantly higher in female than male population, indicating the true difference in the CD4+ T cell values and not the influence of total lymphocyte counts that might vary with the other infection load in the population. The results supported the earlier finding from different population [7,24,27]. It has been suggested that a sex hormone effect could be one possible explanation for the reported gender difference in CD4+ T cell counts [3]. Since the absolute CD4+ T cell count and percentages in male and female populations differ significantly, it was felt appropriate to develop different reference rages for male and female population. We reported difference reference ranges for male and female population as 381-1565 cells/μL and 447-1846 cells/μL respectively.

The absolute CD4 count reported from South India were higher than other geographical region (mean 1039 ± 315, median: 1017 cells/μL) and the CD4 counts from East India were lower as compared to other geographical region (mean 808 ± 255, median: 773 cells/μL) (Table 2). Similar results were obtained for the absolute CD3 counts and percentages. The CD4 counts from north India and west India were comparable. The CD4 counts reported in earlier studies showed a wide range of 321-1800 cells/μL in Western Indians and 304-1864 cells/μL in North Indians [12] which was slightly lower than the counts reported in the present study. Whereas similar values were reported in two studies carried out in eastern region [28,29]. The CD4 percentages reported in Western Indian population in this study were similar to the previous reports [30,31], however the values reported in South Indian population were higher than the previous report [11]. These observations may again be attributed to the differences in the methodologies across the studies.

In case of Eastern region, the absolute CD4+ T cell counts from the participants from Regional Medical Research Centre (RMRC,) Dibrugarh (all are from North East)(Mean: 728 ± 250, median:688 cells/μL) and School of Tropical Medicine, Kolkata (all from either west Bengal or from Orissa)(mean: 888 ± 241, median: 873 cells/μL) differed significantly. Since the CD4 percentages in both these populations were similar (36%), the difference could be attributed to the differences in the absolute lymphocyte counts and not in the CD4+ T lymphocytes. These differences might be due to the environmental, genetic and nutritional factors [32-34] which needs to be confirmed in a larger study within those populations. The study showed no significant influence of alcohol consumption or smoking on the CD4 counts; however the number of habitual alcohol takers in this study was very small. These findings were similar to previous reports [11]. However the economic status; in terms of monthly income, did show significantly higher absolute CD4 counts and CD4 percentages in participants with higher income. This probably indicates the importance of the availability of nutritious food (might be vegetarian or non vegetarian) with higher capacity to pay for it; however this observation needs to be confirmed in a larger study.

The absolute CD4+ T cell count of 250 cells/μL is being used as a cut off value to initiate ART in the National AIDS Control Programme (NACP) in India. In the light of the availability of 'test and treat strategy' in India or provision of ART at higher CD4+ T cell counts in future it is necessary to know the lower reference values in the population as this might influence the outcome of the ART. In conclusion, the present study established the national reference range for CD4 counts in India as 381-1565 cells/μL for male population and 447-1846 cells/μL for female population. The reference values for CD3 counts were 776-2785 cells/μL for Indian male population and 826-2997 cells/μL for female population. The reasons for lower CD4 count in eastern population should be explored in additional study in the target population. The reference ranges could be useful in clinical management of the HIV infection and various immunodeficiencies in India.

## Competing interests

The authors declare that they have no competing interests.

## Authors' contributions

MT designed the study, implemented at the respective site, performed the data analysis and drafted the manuscript. PA designed the questionnaire, developed SOP's for all sites, implemented study and observed quality control at NARI. SA, PB, BB, AJ, KR, RV, MV, KR implemented the study at the respective sites. AD, JM, KN, SP, and RS & AJ carried out the flow cytometry and observed the quality control at the respective sites. RP participated in designing the study and writing the manuscript.

All authors have read and approved the final manuscript.
